# Barrett Esophagus: History, definition and etiopathogeny

**Published:** 2014

**Authors:** C Gindea, R Birla, P Hoara, A Caragui, S Constantinoiu

**Affiliations:** * “Carol Davila” University of Medicine and Pharmacy, Bucharest; “Sf. Maria” Clinical Hospital, General and Esophageal Surgery Department, Bucharest, Romania

**Keywords:** Barrett esophagus, pathogenesis, gastro-esophageal reflux

## Abstract

The injury of the esophageal epithelium may be determined by the reflux of the gastric acid in the esophagus. Barrett's esophagus (BE) is characterized by the replacement of the normal squamous epithelium with the columnar epithelium, when the healing of the lesion occurs. According to some studies, the incidence of the esophageal adenocarcinoma in patients with BE is of about 0,5% per year.

The term Barrett’s esophagus is subjected to interpretation nowadays, so it lacks the clarity needed for the clinical and scientific communication on the subject of columnar metaplasia of the esophageal mucosa.

The major pathogenetic factor in the development of BE is represented by the reflux disease.

The cellular origin of BE is controversial and it represents an issue that needs to be resolved because it will have implications in the putative molecular mechanisms underlying the metaplastic process.

The epigenetic or genetic changes, which alter protein expression, function, and/ or activity, in post-mitotic cells to drive transdifferentiation or in stem/ progenitor cells such that they are reprogrammed to differentiate into columnar rather than squamous cells, are driven by the inflammatory environment created by chronic reflux.

In order to be able to develop better therapeutic strategies for the patients with this disease, an increasing interest in understanding the pathogenesis of BE at the cellular and molecular level presents these days.

## Introduction

In many Western countries, the incidence of esophageal adenocarcinoma has risen more rapidly than in any other cancer over the past four decades [**1**].

If Barrett esophagus (BE) is present, the risque of development esophageal adenocarcinoma is significantly higher. 

BE is an aquired disorder, that occurs due to severe esophageal mucosal injury caused by the chronic gastroesophageal reflux; it is characterized by the replacement of the normal esophagian squamous epithelium with an intestinal columnar epithelium that contains caliciform cells [**2**].

The cellular and molecular mechanisms involved in BE development are still poorly understood as well as the cell origin of BE that still remains controversial. In order to define and identify the cells, which give rise to the metaplastic tissue, several hypotheses have been proposed. 

## History

Even if he was not the first one to describe the condition, Barrett’s esophagus got its name after the Australian-born surgeon, Norman Rupert Barrett, who for most of his career worked as a consultant surgeon at St. Thomas’ Hospital in London.

Barrett described patients with ulcerations in a tubular organ that grossly appeared to be the esophagus, but whose distal, ulcerated portion was lined by columnar epithelium. 

Since Barrett had defined the esophagus as a “part of the foregut, distal to the cricopharyngeal sphincter, which is lined by squamous epithelium”, he argued that the ulcerated, columnar-lined viscus described in those reports was a tubular segment of the stomach that had been tethered within the chest by a congenitally short esophagus.

To support that contention, Barrett noted that the ulcerated columnar lining was always identified as “histological gastric in type” [**3**].

In 1906, Wilder Tileston, a pathologist from Boston, described 3 cases of “peptic ulcer of the oesophagus”, and noted “the close resemblance of the mucous membrane about the ulcer to that normally found in the stomach”. He also wrote, “the first requisite for the formation of the peptic ulcer of the oesophagus is an insufficiency of the cardia” [**4**]. 

In 1953, Allison and Johnstone described 7 patients who presented reflux esophagitis involving an “oesophagus lined with gastric mucous membrane” and refuted Barrett’s contention that the tubular, intra-thoracic, columnar-lined viscus was the stomach [**5**].

Allison and Johnstone’s arguments were eventually accepted by Barrett in a report published in 1957, which suggested that the condition should be called “lower oesophagus lined by columnar epithelium”. The eponym Barrett’s esophagus was kept, whether justified or not [**6**].

The intestinal-type goblet cells in the columnar-lined esophagus were first described in 1951 by Bosher and Taylor [**7**].

In 1952, Morson and Belcher reported the case of a patient who developed an adenocarcinoma in an esophageal mucosa that presented “atrophic changes with a tendency towards intestinal type containing many goblet cells” [**8**]. 

This confusion was somewhat clarified in 1976, when Paull et al. reported a systematic study of 11 patients with Barrett’s esophagus, that by using manometric guidance had esophageal biopsy specimens taken above the lower esophageal sphincter. As many as 3 types of columnar epithelia lining the distal esophagus were found in those patients: 1) a junctional (cardia-type) epithelium that comprised mucus-secreting cells, 2) a gastric fundic type epithelium with parietal and chief cells, and 3) intestinal-type metaplasia, which the authors called specialized columnar epithelium, with prominent goblet cells. The 3 epithelial types were present in different zones in the columnar-lined esophagus, with intestinal-type metaplasia adjacent to squamous epithelium in the most proximal segment, followed by cardia-type epithelium, with gastric fundic-type epithelium lining the most distal esophageal segment [**9**].

In order for the patients to be accepted in studies of Barrett’s esophagus, in the early 1980’s, Skinner et al. chose 3 cm as the extent of esophageal columnar lining required for patients to be enrolled. As a result, endoscopists often dismissed columnar epithelium limited to the distal few centimeters of the esophagus as normal and obtained biopsy specimens to confirm a diagnosis of Barrett’s esophagus only when columnar lining extended some arbitrary distance (e.g. >3 cm) above the gastro-esophageal junction [**10**].

By the late 1980’s, it was well established that adenocarcinoma was associated with the Barrett’s esophagus, so intestinal metaplasia was widely regarded as both the most common type of Barrett’s epithelium and the epithelial type associated with cancer development [**11**].

In 1994 that practice was challenged, when Spechler et al. showed that 18% of consecutive patients in a general endoscopy unit, who presented columnar epithelium that involved <3 cm of the distal esophagus also had intestinal metaplasia. Nevertheless, they showed that symptoms and endoscopic signs of gastro-esophageal reflux disease (GERD) were not reliable markers for intestinal metaplasia in the distal esophagus [**12**].

Both histochemical and genetic studies of cardia-type epithelium have revealed molecular abnormalities, similar to those found in intestinal metaplasia that could predispose patients to carcinogenesis [**13**,**14**]. Recent clinical studies supported the concept that cardia-type epithelium had a malignant potential, so the American Gastroenterology Association (AGA) Institute’s technical review on Barrett’s esophagus recommended that it should now be defined as “the condition in which any extent of metaplastic columnar epithelium that predisposes to cancer development replaces the stratified squamous epithelium that normally lines the distal esophagus.”

## Definition

Nowadays, Barrett’s esophagus is a term that is variably interpreted and lacks the clarity needed for clinical and scientific communication on the topic of columnar metaplasia of the esophageal mucosa [**15**]. It is currently both confusing and ambiguous because of the spectrum of what is currently referred to as “Barrett’s esophagus” that ranges from some clinicians making this diagnosis based only on the endoscopic appearances of any extent, to the requirement that intestinal-type esophageal columnar metaplasia be proven histologically before this diagnosis is made [**16**,**17**].

A consensus was made regarding the terminology that differentiates a purely endoscopic diagnosis of esophageal columnar metaplasia from one that is histologically confirmed.

The endoscopic diagnosis needs confirmation with histology and a term that acknowledges the possibility that the endoscopic appearance may not be diagnostic was chosen: “Endoscopically suspected Barrett’s esophagus” was chosen as an appropriate term, but in the belief that this would be of less concern to patients and their insurers and would prevent patients from being mistakenly labeled as having Barrett’s esophagus, the more neutral, descriptive terminology given in the statement was preferred before the histological confirmation was obtained [**18**].

The Montréal workshop eventually reached the consensus that the label “Barrett's esophagus” should be used when any type of esophageal columnar metaplasia is confirmed histologically, with the qualifier whether intestinal-type metaplasia has been found [**19**].

A consensus-based endoscopic classification system, which determines the C & M criteria to describe and classify endoscopic BE, has been proposed and undergone extensive internal and external validation by trained endoscopists. This system is simple and can be measured reliably by different endoscopists. Central for this classification is the location of the gastroesophageal landmarks, and the validation study described here has shown that these can be reliably identified and located by different endoscopists. The adoption of this standardized classification system may greatly enhance the ability of physicians to gauge the efficacy of treatments for BE in individual patients and the classification of patients with BE in clinical trials. In addition, it may help to better define the natural history of BE [**20**].

**Etiology**

**Gastroesophageal reflux **

The most important pathogenetic factor for the development of BE is showed to be the reflux disease. Because only a minority of patients (around 10–15%) with reflux esophagitis have BE, additional factors that play important roles in determining the apparently sudden development of BE must concur [**21**]. 

Several systemic factors, which may predispose to columnar metaplastic healing of the previously squamous esophageal mucosa, have been identified. Notable among these are the **retinoic acid status** and the effects of genetic heterogeneity, including the expression of differing types of insulin growth factor, and differences between BE patients and controls in CDX2 expression in the squamous mucosa in response to acid and **bile salt-induced injury** [**21**-**23**].

A topic of ongoing research in the pathogenesis of BE development is represented by the bile acid-induced injury. There are three bile acids: cholic acid (CA), chenodeoxycholic acid (CDCA) and deoxycholic acid (DCA). Of these, DCA is the most potent in inducing mucosal injury [**24**].

Their role in BE development was studied on multiple human and animal models. Extracted results from in vivo and in vitro studies demonstrate that bile acids are responsible for the increase of reactive oxygen species (ROS) leading to oxidative DNA damage and activating the NF-κB pathway for cell death [**25**].

The impact of oxidative DNA damage is more severe with bile acids because there is a concomitant decrease in MnSOD expression, which is a scavenger for ROS [**26**].

The harm caused by bile acids can be alleviated with the use of N-acetyl-l-cysteine that strengthens the case for ROS-induced injury. Bile acid also induces cytokine-mediated cell damage and may stimulate the expression of COX2, BMP4 and MUC2 genes for proliferation of intestinal metaplasia [**27**].

**Nitrates**

Nitrate-induced injury is another theory which has recently emerged in the pathogenesis of BE. After a nitrate-rich diet, nitrates are found in abundance in human saliva and are also present in high concentration at the eso-gastric junction [**28**]. 

On contact with acid, nitrates are converted to nitrites and so they become even more carcinogenic. This occurs at the gastroesophageal junction that conforms to the appearance of Barrett’s esophagus at the junctional level. Due to the industrialization of commercial farming, the widespread use of nitrate fertilizers mirrors the increase incidence in esophageal adenocarcinoma [**29**].

In addition, researchers have shown an increased expression of Epidermal Growth Factor Receptor (EGFR) in nitrate-treated esophageal cells in vitro [**30**].

**Obesity** has been known to increase the risk for developing BE, but there is an on-going debate on whether obesity’s contribution comes from visceral adiposity versus the overall increase in body mass index (BMI). IL6 and IL8, cytokines released by adipocytes have been found to be important in the intestinalization process [**31**].

Waist circumference can have some modest independent association with the risk of Barrett's esophagus according to some studies, more than BMI. This finding can represent a partial support for the hypothesis that abdominal obesity increases gastro-esophageal reflux and thus, indirectly the risk for Barrett esophagus [**32**].

One recent study that was made on 309 patients, found that the metabolic syndrome was associated with a 2 fold increase in the risk of the development of Barrett’s esophagus, independent of the presence of gastro-esophageal reflux symptoms. This association is independent of smoking, alcohol use and an increased BMI [**33**].

**Age, race, sex and HH**

Another frequent association with Barrett’s esophagus, together with chronic gastro-esophageal reflux, is represented by an increased age, Caucasian race, male sex and hiatal hernia. A rare finding in children, BE mean age of diagnose in Europe is in the 60s [**34**].

A recognized risk factor for the development of Barrett esophagus is represented by the hiatal hernia and the length of the metaplasia segment is increased in patients with larger hiatal defect. Up to 90% of the patients with BE have an associated hiatal hernia, the latter being responsible, along with the other factors, for the reflux of the gastric juice in the esophagus [**35**].

**Alcohol and smoking**

Some diet and life style factors, like alcohol and smoking seem to be implicated in the development of Barrett esophagus. Latter studies showed that cigarette smoking, besides being implicated in the development of esophageal adenocarcinoma, is also a risk factor for Barrett’s esophagus [**36**].

Alcohol seems not to be involved in the development of Barrett’s esophagus and there are even some studies that found a protective role of moderate wine consumption (<40g/week) in the development of Barrett’s esophagus and esophageal adenocarcinoma [**37**].

**Helicobacter Pylori**

The relationship between Barrett’s esophagus and the infection with Helicobacter Pylori is controversial, even if the bacterial role in the gastric carcinogenesis is well known; most of the studies found a protective role of the bacteria in the development of intestinal metaplasia, probably because of the reduced gastric secretion that it determines [**38**].

**Familial transmission**

Some patients present a familial transmission of BE, some genetic modifications independent of smoking or BMI being observed in the same family [**39**].

**The Cellular Origin of BE**

An issue that needs to be resolved as it will have implications for the putative molecular mechanisms underlying the metaplastic process is represented by the cellular origin of BE.

The inability of researchers to determine the cellular origin of BE is in part due to the inability to observe the process of metaplastic conversion in vivo and the lack of reliable physiological animal models [**40**].

Initially, Barrett's oesophagus was thought to arise as a consequence of the upward cell migration from the transitional zone cells of the gastro-oesophageal junction and it was proposed that these cells would migrate and colonize the distal oesophagus or the gastric cardia, in response to tissue damage from continued toxic exposure to refluxate [**41**].

Furthermore, even if the refluxate-mediated damage of the gastric cells can lead to their upward migration, the different epithelial cell lineages in Barrett's oesophagus still need to be accounted for.

A study conducted on the distribution of oesophageal and gastric cardiac mucosae in oesophagectomy specimens led to the suggestion that cardiac glands may be exposed to the luminal surface and so become columnar epithelial islands which could clonally expand to give rise to Barrett's oesophagus [**42**].

Following the animal studies that indicated that the cell of origin was intrinsic to the esophagus, the initial belief that BE was the result of upward migration of gastric and columnar cells from the gastro-esophageal junction was largely discounted [**43**].

As a result, the predominant theories postulated in the past few decades have been that the metaplastic epithelium arises from a direct conversion of squamous cells to columnar cells, due to a process called transdifferentiation, a term that describes an irreversible metaplastic conversion from one fully differentiated state into another. This hypothesis is supported by a study of the conversion of the murine epithelium from columnar into stratified squamous, during the development of the embryonic oesophagus. So, it was shown that a proportion of cells co-express markers of both squamous (cytokeratin 14) and columnar (cytokeratin 8) differentiation during the columnar–squamous conversion. The researchers suggest that the independence of cell division or apoptosis, squamous oesophageal cells can arise directly from columnar basal cells [**44**].

Therefore, the reverse transformation could account for the switch in phenotype in Barrett's oesophagus. If this switch can occur in adulthood, it remains to be proven. Furthermore, the evidence of new squamous epithelium, which develops after the endoscopic removal of Barrett's oesophagus epithelium (assuming that the differentiated epithelium was completely removed), would weaken the theory of transdifferentiation [**45**].

A related hypothesis suggests that a structure composed of multiple layers of cells that appear squamous in their basal portion and columnar more superficially and express cytokeratins of both squamous and columnar differentiation called an intermediate “multilayered epithelium”, may have also have a role [**46**].

However, recent data have re-ignited the theory that BE results from the upward migration of columnar cells from the gastro-esophageal junction [**47**].

Wang and colleagues examined early esophageal epithelial development in wild-type mouse embryos and, in stark contrast to earlier work by Yu and colleagues, which indicated that the maturation of the columnar epithelium to a squamous epithelium in the developing embryonic esophagus is via transdifferentiation, suggested that the columnar cells lining the embryonic esophagus slough off as squamous epithelial cells migrate down the esophagus to undermine the columnar cells and replace them [**48**]. 

They suggested that a small population of residual embryonic columnar cells are maintained at the squamo-columnar junction in adult mice (and at the gastro-esophageal junction in human adults), and these cells give rise to BE by migrating to replace the proximal squamous epithelium eroded by reflux [**48**].

An attractive theory, the eso-gastric junction cell of origin, may also explain the origin of gastric-type (cardiac- or fundic-type) columnar metaplasia in the distal esophagus. It is unclear whether gastric-type metaplasia is a precursor to intestinal-type metaplasia (BE) or whether they are separate entities that possibly give rise to different adenocarcinoma sub-types [**49**].

An interesting possibility, that there may be more than one cellular origin for BE and that perhaps the progression to eso-gastric adenocarcinoma (EAC) is dependent on which cell type the BE arose from, develops from this.

Another theory suggests that the metaplastic epithelium arises from a change in the commitment (“transcommitment”) of pluripotent stem cells that are responsible for constant refreshing of the esophageal epithelium [**50**]. There are also several possible locations for putative stem cells within the esophagus, including the basal layer of the stratified squamous epithelium, the sub-mucosal glands, or the lining of the ducts of these glands.

The hypothesis that Barrett's oesophagus results from a change in the commitment of multipotent stem cells, which are induced to differentiate from a squamous into a columnar epithelium, as a result of the continuous exposure to environmental stresses such as refluxate, generates an increased interest. The stem cell theory would explain the variety of cellular phenotypes found in Barrett's oesophagus and also the way the regeneration of squamous epithelium after the removal of Barrett's oesophagus is possible, and correlates well with the evidence that the cell of origin is intrinsic to the esophagus [**51**].

The epithelial cells residing in the basal layer of the squamous epithelium represent the first stem cell population that could play a role in this theory. It was observed, in the interfollicular epidermis, which is the most highly characterized squamous epithelium with features similar to the oesophagus, that resident multipotent stem cells give rise to all the different cell lineages present within the adult tissue [**52**].

Therefore, identifying the epithelial stem cells of the squamous oesophagus is the key even if these cells have not been clearly detected and located.

In order to revisit the question of stem cell location, technological developments which allow for a three-dimensional representation of the epithelium by using the epithelial whole mounts and confocal microscopy are now being applied to [**53**,**54**].

A second population of stem cells may be located in the glandular neck region of the esophageal submucosal gland ducts - these ducts are lined in their proximal two-thirds by columnar cells, whereas their distal third is lined with squamous cells, they have been suggested as a location for stem cells responsible for the origin of the columnar epithelium [**55**].

This theory is based on the ulcer-associated cell lineage that occurs next to the areas of ulceration in the gastrointestinal tract, and prefigures a migration of the glandular cells to the surface, through new glands and ducts generated by stem cells in the lamina propria [**56**].

The subject of several studies became the gland duct theory, which seems to support the concept that these cells may be critical for the development of Barrett's oesophagus. RA (retinoic acid) was the first that has been identified as a stimulus for cell differentiation, suggesting that the stromal compartment, which includes the submucosal gland ducts, is the cell source for the columnar cells induced by RA treatment of squamous tissue [**57**].

Bone marrow derived progenitor cells have also been proposed to contribute to BE [**58**].

**BE pathogenesis**

**Transcription factors and morphogens that drive the development of the intestinal epithelium may underlie the development of BE**

Because of the predominance of the transdifferentiation and transcommitment theories, most of the studies have focused on the molecular mechanisms driving the conversion of the squamous epithelium to intestinal-type columnar epithelium.

The inflammatory environment created by chronic reflux induces epigenetic or genetic changes, which alter protein expression, function, and/ or activity in post-mitotic cells to drive transdifferentiation or in stem/ progenitor cells such that they are reprogrammed to differentiate into columnar rather than squamous cells [**59**].

Non-epithelial cells like myofibroblasts, inflammatory cells within the esophageal mucosa and submucosa also influence the development of BE through the release of paracrine factors such as cytokines and other regulatory signals that affect cell differentiation and development within the epithelial layer [**60**].

A number of potential molecular mediators of transdifferentiation or transcommitment in the pathogenesis of BE have been suggested, many of them known to play important roles in the development and homeostasis of normal intestinal epithelium.

**Homeobox Transcription Factors may drive intestinal differentiation in BE**

CDX1 and CDX2, the caudal-related homeobox transcription factors are important in the differentiation of the intestinal epithelium and are known to regulate intestine-specific gene expression. Both are expressed in BE but not present in normal esophagus or gastric epithelial cells [**61**].

CDX2 mRNA is also up regulated in inflamed squamous epithelium (esophagitis) preceding the development of BE [**62**] suggesting that CDX2 may play an initiating role in the development of metaplasia. 

Interestingly, the up-regulation of CDX2 by acid and bile has been shown to occur in esophageal squamous epithelial cells from patients with BE, but not from patients with GERD, but no BE [**63**], perhaps indicating some underlying differences between the patients that may determine the development of metaplasia.

The ectopic expression of CDX1 and CDX2 in squamous esophageal cells in vitro results in the expression of columnar and intestinal differentiation markers such as columnar-type cytokeratins and mucins. When CDX1 was expressed in the esophageal squamous epithelium in an in vitro 3-D culture model, the general morphology of the reconstituted epithelium was still stratified squamous [**64**]. 

Similarly, the ectopic expression of CDX2 in the mouse esophagus did not dramatically alter the morphology of the squamous epithelium, although the basal cells with altered ultra-structural features suggestive of a secretory-cell phenotype were observed, and suggested to be intermediate between squamous and columnar cells [**65**]. 

Likewise, we have shown that the ectopic expression of CDX2 in squamous esophageal epithelium in an in vivo tissue reconstitution model does not alter the differentiation [**66**].

So, the role of CDX1 and CDX2 in the pathogenesis of BE, and the possible mechanism by which they might drive the metaplastic conversion of the squamous epithelium remain unclear.

Several genes from the HOXB family of homeobox domain transcription factors have been implicated in BE. HOXB 5, 6, and 7 are up regulated in BE and the ectopic expression of these genes induces the expression of intestinal differentiation markers K20, villin, and MUC2 in normal esophageal squamous epithelial cells [**67**]. 

Additional studies are required in order to further reveal a possible role of these genes in BE pathogenesis.

**Notch and Wnt signaling pathways may co-operate to promote BE pathogenesis**

The Notch and Wnt signaling pathways, which regulate stem cell maintenance and differentiation in the intestine, have also been implicated in the pathogenesis of BE. Notch signaling is activated in BE crypts, similar to the intestine, and the inhibition of the Notch pathway drives terminal differentiation into goblet cells [**68**].

Notch signaling is activated in the esophageal squamous epithelial cells by bile salts via CDX2 and by ectopic expression of CDX2 in vitro [**69**], suggesting that the activation of this pathway is a possible mechanism by which CDX2 could promote metaplasia. 

Furthermore, the ectopic expression of the downstream efector of Notch signaling, Hath1/Atoh1, induced expression of intestinal- and BE-markers Muc2 and K20 in esophageal squamous epithelial cells in vitro [**65**], further supporting a role for Notch signaling in BE.

However, the activation of Wnt signaling was demonstrated to up-regulate Notch signaling, expression of intestinal differentiation markers, and mucin production in esophageal squamous epithelial cells in vitro [**70**].

This suggests a possible role for Wnt signaling in the pathogenesis of BE, perhaps in concert with Notch.

## Conclusions

A significant risk for the development of adenocarcinoma is represented by the intestinal metaplasia of the esophagus (BE). An increasing interest in understanding the pathogenesis of BE at the cellular and molecular level is shown in order to be able to develop better therapeutic strategies for the patients with this disease. Recent data raise the possibility that the metaplastic epithelium has multiple origins, including cells from the gastro-esophageal junction, so it could explain why only some patients progress to EAC.

Whether the pathogenesis of BE is a process requiring multiple factors, none of which being sufficient on its own to induce complete metaplasia, is still a matter of debate. The intestinal morphogen signaling pathways and transcription factors seem to play important roles in the BE pathogenesis.
